# Prevalence of Self-Reported Anosmia and Ageusia in Elderly Patients Who Had Been Previously Hospitalized by SARS-CoV-2: The LONG-COVID-EXP Multicenter Study

**DOI:** 10.3390/jcm12134391

**Published:** 2023-06-29

**Authors:** César Fernández-de-las-Peñas, Ricardo Ortega-Santiago, Ignacio Cancela-Cilleruelo, Jorge Rodríguez-Jiménez, Stella Fuensalida-Novo, José D. Martín-Guerrero, Óscar J. Pellicer-Valero, Margarita Cigarán-Méndez

**Affiliations:** 1 Department of Physical Therapy, Occupational Therapy, Physical Medicine and Rehabilitation, Universidad Rey Juan Carlos (URJC), 28922 Alcorcón, Spain; cesar.fernandez@urjc.es (C.F.-d.-l.-P.); ignacio.cancela@urjc.es (I.C.-C.); jorge.rodriguez@urjc.es (J.R.-J.); stella.fuensalida@urjc.es (S.F.-N.); 2 Intelligent Data Analysis Laboratory, Department of Electronic Engineering, ETSE (Engineering School), Universitat de València (UV), 46100 Burjassot, Spain; jose.d.martin@uv.es; 3 Valencian Graduate School and Research Network of Artificial Intelligence (ValgrAI), 46022 València, Spain; 4 Image Processing Laboratory (IPL), Universitat de València, Parc Científic, 46010 València, Spain; oscar.pellicer@uv.es; 5 Department of Psychology, Universidad Rey Juan Carlos (URJC), 28922 Alcorcón, Spain; margarita.cigaran@urjc.es

**Keywords:** COVID-19, anosmia, ageusia, symptoms, trajectory, Sankey plots

## Abstract

We explored two different graph methods for visualizing the prevalence of self-reported post-COVID anosmia and ageusia in a large sample of individuals who had been previously hospitalized in five different hospitals. A cohort of 1266 previously hospitalized COVID-19 survivors participated. Participants were assessed at hospitalization (T0) and at three different follow-up periods: 8.4 (T1), 13.2 (T2), and 18.3 (T3) months after hospital discharge. They were asked about the presence of self-reported anosmia and ageusia that they attributed to infection. Anosmia was defined as a self-perceived feeling of complete loss of smell. Ageusia was defined as a self-perceived feeling of complete loss of taste. Data about hospitalization were recorded from medical records. The results revealed that the prevalence of anosmia decreased from 8.29% (*n* = 105) at hospitalization (T0), to 4.47% (*n* = 56) at T1, to 3.27% (*n* = 41) at T2, and 3.35% (*n* = 42) at T3. Similarly, the prevalence of ageusia was 7.10% (*n* = 89) at the onset of SARS-CoV-2 infection (T0), but decreased to 3.03% (*n* = 38) at T1, to 1.99% (*n* = 25) at T2, and 1.36% (*n* = 17) at T3. The Sankey plots showed that only 10 (0.8%) and 11 (0.88%) patients exhibited anosmia and ageusia throughout all the follow-ups. The exponential curves revealed a progressive decrease in prevalence, demonstrating that self-reported anosmia and ageusia improved in the years following hospitalization. The female sex (OR4.254, 95% CI 1.184–15.294) and sufferers of asthma (OR7.086, 95% CI 1.359–36.936) were factors associated with the development of anosmia at T2, whereas internal care unit admission was a protective factor (OR0.891, 95% CI 0.819–0.970) for developing anosmia at T2. The use of a graphical method, such as a Sankey plot, shows that post-COVID self-reported anosmia and ageusia exhibit fluctuations during the first years after SARS-CoV-2 infection. Additionally, self-reported anosmia and ageusia also show a decrease in prevalence during the first years after infection, as expressed by exponential bar plots. The female sex was associated with the development of post-COVID anosmia, but not ageusia, in our cohort of elderly patients previously hospitalized due to COVID-19.

## 1. Introduction

After three years of the pandemic, it is known that coronavirus disease 2019 (COVID-19) can be considered a multiorgan disease affecting different systems [1]. The affectation of multiple systems facilitates the presence of heterogeneous symptomatology at the acute phase. For instance, fever, cough, and/or dyspnea are the symptoms most commonly experienced during the COVID-19 acute phase [2]. In addition to respiratory symptomatology, neurological symptoms, including headache, ageusia, or anosmia, are also prevalent during the acute phase of a severe acute respiratory syndrome coronavirus 2 (SARS-CoV-2) infection. In fact, loss of smell (anosmia) and taste (ageusia) had a prevalence of up to 62% at the onset phase of COVID-19 disease and are used to predict symptoms of the SARS-CoV-2 infection [3]. The Cochrane review has suggested that symptoms, such as anosmia and/or ageusia, may be useful for identifying the presence of COVID-19 since they exhibit specificities over 90% (anosmia: 94.2%, 95% CI 90.6–96.5%; ageusia: 92.6%, 95% CI 83.1–97%) [4]. Additionally, the presence of ageusia or anosmia as an onset-associated symptom has been associated with mild, but not severe, COVID-19 disease [5]. This association of a more favorable clinical course of COVID-19 can be related to the fact that subjects with SARS-CoV-2 infection reporting olfactory/gustatory disorders exhibited lower inflammatory responses than those without these chemosensory alterations [6].

Importantly, several patients experience long-lasting symptoms even when the acute infection has passed, and a condition called long-COVID can be present several months or years after the acute infection [7]. There is no consensus on the definition or the use of the term long-COVID. A Delphi study proposed the term post-COVID-19 condition and the following definition: “Post-COVID-19 condition occurs in people with a history of probable or confirmed SARS-CoV-2 infection, usually three months from the onset of COVID-19 disease with symptoms that last for at least two months and cannot be explained by an alternative medical diagnosis” [8]. The literature describes the presence of more than 100 symptoms that can be long-lasting after an acute SARS-CoV-2 infection [9]. The Global Burden of Disease Long COVID study (*n* = 1.2 million of symptomatic COVID-19 survivors) reported that 51% experienced the presence of at least one post-COVID symptom in the first months after the SARS-CoV-2 acute infection [10]. As expected, long-lasting ageusia and anosmia can also be present after the acute phase. Different meta-analyses have observed that the prevalence of post-COVID anosmia and ageusia can range from 12% to 20% [11,12,13]. Although smell and taste disorders are post-COVID symptoms not as bothersome as others, e.g., fatigue or dyspnea, the presence of ageusia and anosmia may lead to disruption to basic daily living activities impacting the well-being, physical health, and personal relationships of a patient [14,15]. Preliminary data suggests that 80% of patients may expect a spontaneous recovery of smell and taste disorders two [16] or six [17] months after an acute infection. However, Tan et al., found, by creating a parametric cure meta-analytic modeling, that a substantial proportion of individuals who had survived an acute SARS-CoV-2 infection could develop long-lasting (up to six months after) smell or taste disorders [18]. 

Importantly to remark is that most studies investigating the prevalence of ageusia or anosmia have mainly used cross-sectional designs by assessing the presence of these symptoms just once or twice and also had commonly used follow-up periods no longer than six months after infection [11,12,13,18]. A previous study (the LONG-COVID-EXP) analyzed the evolution of ageusia and anosmia from the onset of the infection up to the first year after a SARS-CoV-2 infection in a cohort of previously hospitalized COVID-19 survivors [19]. Understanding the longitudinal trajectory of post-COVID anosmia and ageusia could have significant implications in early diagnosis of COVID-19, triaging of patients at emergency departments, and management of individuals with long-COVID. We present the complete follow-up analysis of the LONG-COVID-EXP study by using exponential bar plots for visualizing the evolution of ageusia and anosmia from the onset of the acute infection, up to 6, 12, and 18 months after hospitalization. Additionally, Sankey plots were used as a novel graph method for visualizing the fluctuating evolution of post-COVID ageusia and anosmia. A secondary aim is to identify the potential risk factors associated with the development of post-COVID anosmia and ageusia at long-term follow-up. 

## 2. Methods

### 2.1. Participants

The LONG-COVID-EXP-CM is a multicenter study including a cohort of individuals who had been hospitalized because of a SARS-CoV-2 acute infection during the first wave of the pandemic (from 10 March to 31 May 2020) in five urban public hospitals in Madrid (Spain). To be included, SARS-CoV-2 infection should have been diagnosed at hospital admission by real-time reverse transcription–polymerase chain reaction (RT–PCR) assay of nasopharyngeal or/oral swab samples and the presence of radiological changes. As previously described, from all subjects hospitalized in the five participating hospitals during the first wave of the outbreak in the involved hospitals (*n* = 7150), a sample of 400 individuals from each hospital was randomly selected by an online software providing an initial sample of 2000 participants. The Ethics Committee of all hospitals approved the study (HUFA20/126, HUF/EC1517, HSO25112020, HUIL/092-20, HCSC20/495E). Participants provided their verbal informed consent before any data was collected. 

### 2.2. Procedure

The procedure of this multicenter cohort study has been previously described [19]. Briefly, clinical and hospitalization data were collected from hospital medical records. Participants were scheduled for a telephone interview conducted by trained healthcare professionals at three different follow-up periods separated six months each after hospitalization—T1, T2, and T3. At the interview, participants were asked about self-reported anosmia and ageusia. Anosmia was defined as a self-perceived feeling of complete loss of smell, whereas ageusia was defined as a self-perceived feeling of complete loss of taste. We specifically asked for anosmia or ageusia that the individuals attributed to COVID-19. 

### 2.3. Sankey Plots

Visualization of the flow and evolution of patients in relation to the presence/absence of ageusia or anosmia over time was provided by using a Sankey plot [20]. In a Sankey plot, the X axis represents each follow-up (COVID-19 onset, six, twelve, eighteen months after), while the Y axis represents the percentage of subjects with or without each symptom (e.g., anosmia, ageusia). The darker vertical bars (called nodes in a Sankey plot) represent the percentage of subjects with or without symptoms at that particular follow-up. The arcs graph the flows (that is, the change) of subjects between the state of positive/negative in relation to each symptom. The percentage of subjects (from the total sample) is proportional to the width of that arc. The percentage of individuals reporting or not the symptom is placed on the right side of the vertical bar, whereas the flows with the percentage of individuals that they contain are annotated into the left side of the vertical bar [20].

### 2.4. Exponential Bar Plots

The Matplotlib 3.3.4 program (https://matplotlib.org/) was used to create the exponential bar plots. They were fitted to the following formula $y=Ke^{ct}$, where y is the model proposing the prevalence of each symptom (fatigue or dyspnea) at a time t (in months), and K and c are the proposed parameters of the model as previously published [19].

### 2.5. Statistical Analysis

Finally, variables collected at hospital admission (COVID-19 onset follow-up, T0) and at the first follow-up period (T1, six months) were entered into multivariate logistic regressions to identify their association with the development of post-COVID anosmia or ageusia at T2 and T3 time points. Analyses were conducted with Python’s library statsmodels 0.11.1. Adjusted odds ratios (OR) with their respective confidence intervals (95% CI) are summarized. A priori, the level of significance was set at 0.05.

## 3. Results

From a cohort of 2000 subjects randomly selected from all the involved hospitals to participate, a total of 1969 (46.5% women, age: 61, SD: 16 years old) were included at baseline (T0) and 6 months (T1); 1593 were evaluated at 12 months (T2) and 1266 at 18 months (T3). Thus, final analyses were conducted on the sample (*n* = 1266, 64.3% from the original), completing all time point follow-ups: T1 (mean: 8.4, SD: 1.5), T2 (mean: 13.2, SD: 1.0) and T3 (mean: 18.3, SD: 1.0) months after hospital discharge. Table 1 shows the onset symptoms of the acute infection (hospital admission) and the medical data of the sample.

**Figure 1:**
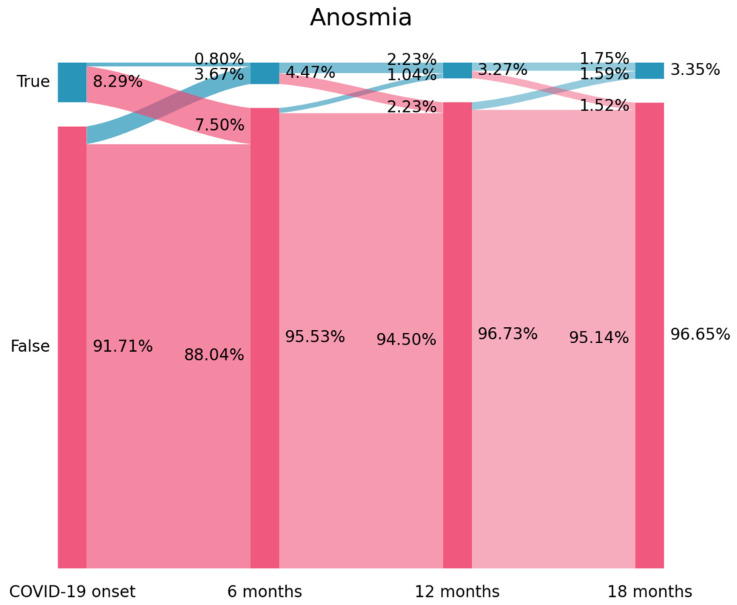
Prevalence of self-reported anosmia (from left to right) at T0 (hospital admission, COVID-19 onset), T1 (8.4 months after), T2 (13.2 months after), and T3 (18.3 months after) as depicted by Sankey plots.

The prevalence of self-reported anosmia was 8.29% (*n* = 105) at hospitalization (T0) and decreased to 4.47% (*n* = 56) at T1, to 3.27% (*n* = 41) at T2 and 3.35% (*n* = 42) at T3 (Figure 1). Looking at Figure 1, 91.3% of those subjects (*n* = 95/105) experiencing anosmia at the onset of the infection (T0) had recovered six months after (7.5% arc from true at T0 to false at T1). In fact, 81.1% (*n* = 46/56) of individuals reporting anosmia at T1 can be considered that they developed “new onset” post-COVID anosmia since they did not report this symptom at the acute phase of the infection (3.67% arc from false at T0 to true at T1). A similar tendency was seen between T1–T2 and T2–T3 follow-up periods but with a small number of subjects. 

For instance, 11 subjects not experiencing anosmia at T1 reported the presence of this symptom at T2 point (1.04% arc from false at T1 to true at T2). The Sankey plot graphed that only 10 patients (0.8% of the sample) self-reported anosmia as a COVID-19 associated-symptom from the acute infection (hospital admission-T0) and throughout all the follow-up periods.

**Figure 2:**
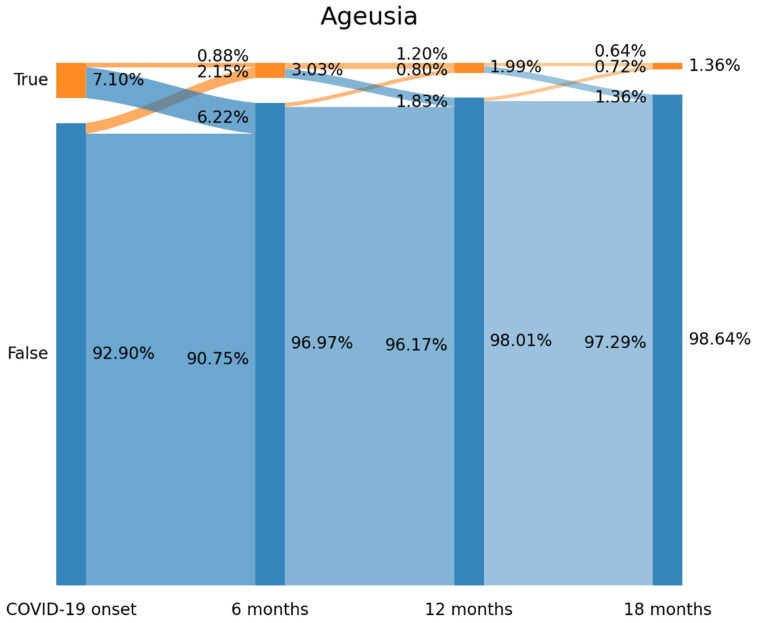
Prevalence of self-reported ageusia (from left to right) at T0 (hospital admission, COVID-19 onset), T1 (8.4 months after), T2 (13.2 months after), and T3 (18.3 months after) as depicted by Sankey plots.

The prevalence of ageusia decreased from 7.10% (*n* = 89) at the onset of SARS-CoV-2 infection (T0), to 3.03 (*n* = 38) at T1, to 1.99% (*n* = 25) at T2 and 1.36% (*n* = 17) at T3 (Figure 2). Figure 2 revealed that 87.6% of those subjects (*n* = 78/89) experiencing ageusia at the onset of COVID-19 (T0) recovered at T1 (6.22% arc from true at T0 to false at T1). Therefore, 71% (*n* = 27/38) of individuals reporting this symptom at T1 developed “new-onset” post-COVID ageusia since they did not report ageusia at COVID-19 onset (2.15% arc from false at T0 to true at T1). A similar tendency was seen between the remaining follow-up periods but with a small number of individuals. The Sankey plot visualized that only 11 patients (0.88% of the sample) self-reported ageusia as a symptom of the acute infection throughout all the follow-up periods.

**Figure 3:**
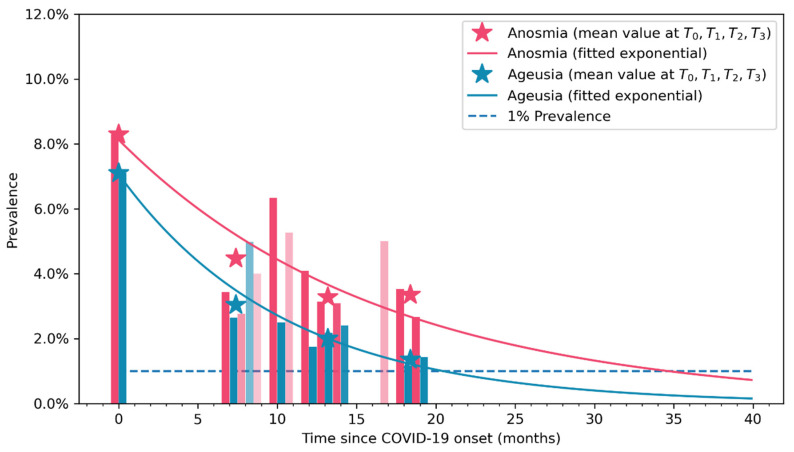
Prevalence of self-reported anosmia (in light red) and ageusia (in light blue) symptoms trajectory as visualized by exponential bar curves. Opacity approximately indicates the sample size at that follow-up time. Asterisks represent mean values at T0, T1, T2, and T3 follow-ups.

The exponential curves graphing the longitudinal evolution of anosmia and ageusia are depicted in Figure 3. The fit model reveals a naturally decreased prevalence trend in both anosmia and ageusia during the following three years after the infection. Vertical bars represent the percentage of individuals that reported anosmia (light red) or ageusia (in light blue) at each follow-up period. The asterisks represent the point prevalence value at each moment (T0, T1, T2, T3) in the graph. 

The multivariate regression models revealed that female sex (OR 4.254, 95% CI 1.184 to 15.294, *p* = 0.027) and suffering from asthma as medical co-morbidity before the infection (OR 7.086, 95% CI 1.359 to 36.936, *p* = 0.02) were factors associated with the development of anosmia at T2 follow-up period whereas having admitted to internal care unit (ICU) was a protective factor (OR 0.891, 95% CI 0.819 to 0.970, *p* = 0.007) for developing anosmia at T2 follow-up period. No variable was associated either with the development of anosmia at the T3 follow-up period or the development of ageusia at any post-COVID follow-up period. 

## 4. Discussion

This is the first cohort study using two approaches for visualizing the trajectory of anosmia and ageusia COVID-19-associated symptoms during the first years after the infection in people who had been previously hospitalized because of COVID-19. Sankey plots revealed a fluctuating evolution of anosmia and ageusia during the first years after the infection, revealing that almost 90% of subjects reporting these symptoms at COVID-19 onset spontaneously recovered. In accordance, the exponential bar plots also visualized a decrease in the prevalence of post-COVID self-reported anosmia and ageusia during the first years after the infection.

Previous meta-analyses, including cross-sectional studies of individuals infected during the first wave of the pandemic, reported an overall prevalence of post-COVID anosmia and ageusia ranging from 12% to 20% during the first six months after infection [11,12,13]. Similarly, the Global Burden of Disease Long COVID study [10] reported a prevalence of anosmia of 12.2% (95% CI 7.7–16.6%) and a prevalence of 11.7% (95% CI 6.1–17.3%) for ageusia. Prevalence rates of self-reported anosmia and ageusia reported in our study (1–5%) were slightly inferior to previous meta-analyses [10,11,12,13] but agree with a meta-analytic recovery model suggesting that persistent smell or taste dysfunction might be developed by up to 5% of patients [18]. Several explanations, including different study designs (longitudinal vs. cross-sectional), follow-up periods (one, two, three, nine months), collection procedure (phone interview, face-to-face), or use of self-reported data or objective assessment, could explain the heterogeneous prevalence among studies. In fact, evidence has shown a wide variability of chemosensory impairments prevalence according to subjective self-reported reports or objective testing [22]. Another important difference is the inclusion of a sample of hospitalized patients. Published data support that the prevalence of anosmia and ageusia is lower in hospitalized than in non-hospitalized patients [12,23]. In addition, anosmia and ageusia are more frequently experienced by mid-aged patients (40–50 years), and the age in our sample was slightly older. These differences may explain the lower prevalence rates observed in our study. 

The exponential model graphed that the prevalence of anosmia and ageusia as post-COVID symptoms decreased; accordingly, long-term follow-up periods will also lead to lower prevalence rates. Our results agree with the meta-analysis by Tan et al., reporting a progressive decrease prevalence of anosmia and ageusia during the first six months after SARS-CoV-2 infection [18]. Additionally, Tan et al., also observed that between 70% and 90% of COVID-19 survivors recovered their sense of smell or taste during the first six months after the infection [18]; results were also observed in our study in both exponential and Sankey plots. 

The use of Sankey plots permits visualization of the fluctuating nature of post-COVID symptoms in the same patient, as previously suggested [24]. In fact, Sankey plots were able to identify the following scenarios according to the presence of a symptom at the acute phase of infection or just at the post-COVID phase [25] in the development of anosmia and ageusia:

1. New-onset post-COVID anosmia or ageusia: subjects experiencing anosmia/ageusia after the infection but not at the acute phase (3.67% arc from false at T0 to true at T1 on Figure 1 for anosmia and 2.15% arc from false at T0 to true at T1 on Figure 2 for ageusia);
2. Persistent post-COVID anosmia or ageusia: patients starting with anosmia/ageusia as an onset symptom of the infection and experiencing the symptom(s) throughout all the follow-ups (0.80% of the sample for anosmia in Figure 1 and 0.88% for ageusia in Figure 2).

By definition, new-onset and persistent post-COVID anosmia/ageusia are attributable to SARS-CoV-2 if they appear no later than three months after onset [8]; nevertheless, we observed that some patients self-reported the presence of anosmia or ageusia at T2 but not at T1, that is, more than 8 months after the infection (1.04% arc from false at T1 to true at T2 on Figure 1 for anosmia and 0.80% arc from false at T1 to true at T2 on Figure 2 for ageusia). This post-COVID symptom was described as a “delayed onset post-COVID symptom” [25]; however, this third scenario is more difficult to attribute to SARS-CoV-2 since the post-COVID symptom appears several months after. It is possible that other factors (e.g., post-traumatic stress, medical co-morbidities, reinfections) are more related to the development of this “delayed” post-COVID symptom rather than the acute initial infection. 

Post-COVID anosmia or ageusia may arise from a combination of biological factors, e.g., affection of the angiotensin-converting enzyme II receptor (ACE-2) receptors, the long-term damage of the olfactory epithelium cells, affection of the frontal lobe, or inflammatory obstruction of olfactory clefts [26,27]. It is also possible that individuals developing long-term chemosensory disorders can exhibit a particular viral mechanism, e.g., viral persistence in the olfactory nerve or the brain. Thus, the identification of risk factors associated with the development of anosmia and/or ageusia may help to manage these post-COVID symptoms [28], albeit they tend to recover spontaneously [29]. To date, limited data on specific factors associated with the development of post-COVID anosmia or ageusia is available [30]. Female sex is a risk factor clearly associated with overall post-COVID-19 conditions in the former literature [31]. We also identified that the female sex was a risk factor significantly associated with the development of post-COVID anosmia, but not ageusia. In agreement with our results, Tan et al., also observed that the female sex was associated with poorer recovery of both smell and taste than the male sex [18]. We also found a higher prevalence of post-COVID anosmia in individuals with pre-existing asthma, which could be an expected finding. However, it should be recognized that other conditions not investigated in the current study, such as rhinitis, could be more relevant to the development of anosmia. Interestingly, being admitted to ICU was a protective factor for post-COVID anosmia, which could be associated with the fact that anosmia and ageusia are much less prevalent in hospitalized COVID-19 survivors [12,23]. This finding would agree with current knowledge that COVID-19 disease severity is not associated with the overall development of long-COVID [32]. It would also be possible that intensive treatment provided at the ICU during the acute phase could help to avoid the development of chemosensory dysfunction, even in critical patients. What treatment is the most effective in preventing chemosensory dysfunction would be an interesting question to be answered in future studies. 

Although this multicenter cohort study used two different methods of visualization and analysis of post-COVID anosmia and ageusia, some limitations should also be recognized. First, the cohort included individuals who had been hospitalized because of an acute SARS-CoV-2 infection. Current results should not be extrapolated to non-hospitalized individuals where the prevalence of anosmia and ageusia is higher. Second, data were self-reported and collected throughout telephonic interviews, a procedure with an inherent bias. However, the use of telephonic interviews is a feasible way to assess large cohorts like that one (over 1000 patients during more than one-year follow-up). Third, since symptoms were self-reported, other smell/taste disturbances, such as hyposmia (reduced smell) or dysgeusia (distortion of basic tastes such as salt, sweet, sour, and bitter), were not assessed. Finally, although we asked for specific treatments received by the patients, most of them answered that they had not received olfactory treatment during the follow-up period. In fact, the Cochrane review found limited evidence for the effectiveness of treatments for persistent olfactory dysfunction following COVID-19 infection but identified a number of ongoing trials evaluating the effects of different olfactory treatments for managing post-COVID anosmia [33].

## 5. Conclusions

This study identified a fluctuating evolution of post-COVID self-reported anosmia and ageusia during the first years after an acute SARS-CoV-2 infection in a cohort of subjects who had been previously hospitalized, as depicted by the use of Sankey plots as a visualization method. Exponential bar plots also visualized a decreased trend of anosmia and ageusia in the first years after hospitalization. Female sex was a risk factor associated with the presence of post-COVID anosmia, but not ageusia. It should be considered that self-reported ageusia most likely can be translated to a loss of flavor, as there is no convincing data on loss of taste function confirmed by psychophysical assessment after COVID-19.

## Figures and Tables

**Table 1:** Demographic and clinical data of the sample (*n* = 1266) [21].

Age, mean (SD), years	61 (16.5)
Female (%)	578 (45.6%)
Weight, mean (SD), kg.	74.5 (14.5)
Height, mean (SD), cm.	165 (19.0)
COVID-19 symptoms at hospital admission, *n* (%)—T0	
Fever	948 (74.9%)
Dyspnea	361 (28.5%)
Myalgia	374 (29.5%)
Cough	360 (28.4%)
Headache	135 (16.7%)
Diarrhea	105 (8.3%)
Anosmia	105 (8.3%)
Ageusia	66 (7.0%)
Throat Pain	66 (5.2%)
Vomiting	39 (3.0%)
Medical co-morbidities	
Hypertension	336 (26.5%)
Other (Cancer, Kidney Disease)	207 (16.3%)
Diabetes	158 (12.5%)
Cardiovascular Disease	141 (11.2%)
Asthma	85 (6.7%)
Obesity	57 (4.5%)
Chronic Obstructive Pulmonary Disease	47 (3.7%)
Rheumatological Disease	16 (1.3%)
Stay at the hospital, mean (SD), days	10.5 (10.8)
Intensive Care Unit (ICU) admission	78 (6.2%)

## Data Availability

All data are presented in the text.
